# Proteomic analysis of the serum in dogs with pulmonary hypertension secondary to myxomatous mitral valve disease: the preliminary study

**DOI:** 10.3389/fvets.2024.1327453

**Published:** 2024-03-26

**Authors:** Siriwan Sakarin, Anudep Rungsipipat, Sittiruk Roytrakul, Janthima Jaresitthikunchai, Narumon Phaonakrop, Sawanya Charoenlappanit, Siriwan Thaisakun, Sirilak Disatian Surachetpong

**Affiliations:** ^1^Faculty of Veterinary Science, Department of Veterinary Medicine, Center of Excellence for Companion Animal Cancer, Chulalongkorn University, Bangkok, Thailand; ^2^Faculty of Veterinary Science, Department of Pathology, Chulalongkorn University, Bangkok, Thailand; ^3^Functional Proteomics Technology Laboratory, National Center for Genetic Engineering and Biotechnology (BIOTEC), National Science and Technology Development Agency, Pathum Thani, Thailand

**Keywords:** biomarkers, dogs, myxomatous mitral valve disease, proteomics, pulmonary hypertension, serum

## Abstract

**Background:**

Pulmonary hypertension (PH) is a common complication in dogs with myxomatous mitral valve disease (MMVD), characterized by elevated blood pressure in pulmonary artery. Echocardiography is a reliable technique for PH diagnosis in veterinary medicine. However, it is limited to use as an early detection method. Liquid chromatography–tandem mass spectrometry (LC–MS/MS) has found extensive application in the discovery of serum protein biomarkers for various diseases. The objective of this study was to identify serum proteins in healthy control dogs and MMVD dogs both with and without PH using LC–MS/MS.

**Materials and methods:**

In this research, a total of 81 small-breed dogs participated, and they were categorized into three groups: the control (*n* = 28), MMVD (*n* = 24) and MMVD+PH (*n* = 29) groups. Serum samples were collected and analyzed by LC–MS/MS.

**Results:**

Differentially expressed proteins were identified, and the upregulated and downregulated proteins in MMVD+PH group including Myomesin 1 (MYOM1) and Histone deacetylase 7 (HDAC7), Pleckstrin homology domain containing M3 (PLEKHM3), Diacylglycerol lipase alpha (DAGLA) and Tubulin tyrosine ligase like 6 (TTLL6) were selected as proteins of interest in MMVD dogs with PH.

**Conclusion:**

Different types of proteins have been identified in healthy dogs and MMVD dogs with and without PH. Additional studies are needed to investigate the potential of these proteins as biomarkers for PH in dogs with MMVD.

## Introduction

Pulmonary hypertension (PH) is characterized by increased pressure in the pulmonary arteries (PA). This condition can occur in dogs, particularly those with myxomatous mitral valve disease (MMVD) (1), which is the primary contributor to PH in dogs. MMVD is a prevalent acquired heart condition in small-breed dogs, leading to the mitral valve thickening and regurgitation (2, 3). The pathogenesis of PH in MMVD has not been fully described, but the following mechanism has been proposed for the development of PH in dogs with MMVD: the thickened mitral valve fails to close completely during systole, resulting in regurgitant flow, which elevates left atrial pressure and causes pulmonary vein congestion and pulmonary oedema. In response to chronic pulmonary oedema, vasoconstriction and remodeling of the PA may occur, ultimately contributing to PH (4). The occurrence of PH in dogs with MMVD is approximately 14–53%. Myxomatous mitral valve disease dogs that developed PH have a worse prognosis and shorter median survival time than dogs without PH (5).

The diagnosis of PH in veterinary medicine has been based on clinical signs suggestive of PH, such as syncope, dyspnoea, exercise intolerance and ascites, as well as echocardiographic signs of PH and estimated pulmonary arterial pressure (PAP) determined by echocardiography (1). However, several studies in humans (6), animal models (7) and dogs (8) suggest that pulmonary arterial remodeling, particularly medial thickening, the major structural change of the PA in PH (3, 9), may manifest before the detection of elevated PAP. For this reason, detection of increased PAP by echocardiography may be too late, and pulmonary arterial remodeling may become irreversible. There is no specific drug that can cure PH in MMVD dogs; however, previous studies suggest that medial thickening due to proliferating smooth muscle cells can be reversed during the initial stages of PH (10, 11). Therefore, early detection and treatment could slow disease progression, improve outcomes and prolong survival.

As described previously, dogs with MMVD and suspected of developing PH are usually diagnosed by echocardiography, which is of limited utility as an early detection method. In addition, this technique has some limitations, such as the lack of patient cooperation during the procedure and the need for experienced ultrasonographers and expensive ultrasound equipment. Therefore, other methods that can detect PH at an early stage are needed. Biomarker measurement is of interest as a possible alternative method for early detection of various diseases (12). N-terminal pro B-type natriuretic peptide (NT-proBNP) and cardiac troponin I (cTnI) have become significant in the veterinary cardiovascular field (13). However, these proteins are mainly used as additional diagnostic tools for MMVD (14) and are insufficient as biomarkers to detect PH in dogs (15–17). At present, there is no established biomarkers for the diagnosis of PH in dogs.

Liquid chromatography tandem-mass spectrometry (LC–MS/MS) is an interesting technique widely used to discover potential protein biomarkers in various diseases, including PH (18, 19). In people with PH, several studies have used LC–MS/MS to search for PH specific proteins. Those studies found that proteins such as complement 4a des Arg (C4a), leucine-rich α-2-glycoprotein (LRG), complement component 3 (C3), RAF proto-oncogene serine/threonine-protein kinase (RAF1) and insulin-like growth factor binding protein (IGFBP) were diagnostic biomarkers for idiopathic PH (20–22). Although several biomarker discovery studies have been published in people with PH, there is only one study investigating potential protein candidates in PH dogs secondary to MMVD (23). However, that study was preliminary and was conducted with a small sample size without age-matched controls; additional studies with more samples are needed. Therefore, this study aimed to identify serum proteins in healthy control dogs and MMVD dogs, both with and without PH using LC–MS/MS. We hypothesized that MMVD dogs with PH would exhibit distinct protein expression compared to those without PH and healthy control dogs. The proteins showing differential expression could be identified through LC–MS/MS.

## Materials and methods

### Animals

Serum samples were obtained from older small breed dogs, older than 7 years and weighing less than 10 kg, presented to the Small Animal Hospital, Faculty of Veterinary Science, Chulalongkorn University, Thailand. The owners obtained informed consent before enrolling their dogs in the study. All procedures were conducted followed by the recommendations in the ARRIVE guidelines. The Institutional Animal Care and Use Committee, Faculty of Veterinary Science, Chulalongkorn University approved the protocol for the study (Approval number, 1831099; Approval date, 4 February 2019). On the day that blood is being collected, all dogs underwent a history assessment, physical examination, blood pressure measurement, chest radiography, electrocardiography, and echocardiography. Dogs with other cardiovascular disease, pulmonary disease, heartworm infestation, systemic hypertension, neoplasia, or systemic disease that may cause PH or affect serum protein expression were excluded.

Systolic blood pressure was measured three times with an oscillometer and then calculated as mean systolic blood pressure. For assessment of cardiac rhythm, a standard six-lead electrocardiogram was recorded with the dogs placed in the right lateral position. Thoracic radiographs were obtained in ventrodorsal and right lateral views of all recruited dogs to detect signs of cardiomegaly, left-sided and/or right-sided cardiac enlargement, congestion of pulmonary veins, pulmonary artery enlargement, pulmonary oedema and pulmonary parenchymal abnormalities. An Echocardiography was conducted by a skilled sonologist using an ultrasound machine (M9, Mindray, SZ, China) with a 4–12 MHz phased array transducer to confirm the diagnosis of MMVD, determine the structural changes of the heart and vessels and measure the tricuspid regurgitation (TR) peak velocity to calculate the estimated PAP. All dogs were confined to position on their right and left sides without the use of sedation. The enrolled dogs were subsequently separated into three groups: the control (*n* = 28), MMVD (*n* = 24) and the MMVD with PH (MMVD+PH) groups (*n* = 29).

The control group comprised 28 healthy dogs with no past record or clinical indications of cardiorespiratory disease. Physical examination revealed normal heart and lung sounds. No signs of systemic hypertension or cardiac arrhythmias were noted. Chest radiography and echocardiography were conducted to confirm that dogs in this group had normal cardiac and pulmonary status. Twenty-four dogs diagnosed with MMVD stage C were assigned to the MMVD group. All dogs exhibited past or current symptoms of heart failure, such as cough, exercise intolerance or dyspnea, as determined by history taking, physical examination and radiographic evidence of pulmonary oedema. In order to recruited into this group, dogs required to exhibit radiographic evidence of cardiomegaly with VHS of >10.5 (24) and echocardiographic findings of the thickened mitral valve and regurgitation, left atrial and left ventricular enlargement defined as the ratio of the left atrial to aorta (LA/AO) dimension of ≥1.6 and a normalized left ventricular internal diameter at the end of diastole (LVIDd) of ≥1.7 (5, 25). According to the American College of Veterinary Internal Medicine (ACVIM) consensus guidelines for the diagnosis, classification, treatment, and monitoring of PH dogs (1), twenty-nine dogs with MMVD stage C and an intermediate to high probability of PH were included in the MMVD+PH group. The probability of PH was classified based on the TR peak velocity assessed by spectral Doppler echocardiography and anatomic signs of PH, including enlargement of the right ventricle and atrium, pulmonary artery, and caudal vena cava evaluated using two-dimensional echocardiography. Dogs with a peak TR velocity exceeding 3 m/s, along with either no anatomic signs or just one anatomic sign of PH, were categorized as having an intermediate probability of PH. In contrast, dogs meeting the criteria of a peak TR velocity above 3 m/s and two or more anatomic signs of PH, or a peak TR velocity exceeding 3.4 m/s with at least one anatomic sign of PH, were classified as having a high probability of PH (1). To calculate the estimated PAP, the simplified Bernoulli equation was used as follows: Pressure gradient = 4 x peak TR velocity^2^ (m/s) (1).

### Collecting and processing of samples

Prior to the blood sampling, every dog abstained from food for a minimum of 4 h to reduce the effects of the meal on serum protein concentration (26). Three milliliters of blood were collected early in the morning by venipuncture through either the cephalic or saphenous vein with a 21-gauge needle and divided into 1 mL for routine hematological and blood chemistry analysis, and the remaining 2 mL for proteomic analysis. Because the pre-analytical steps such as sample collection, sample preparation and sample storage may have an impact on the sensitivity, selectivity, reproducibility and recovery of reliable biomarkers (27, 28), a single individual conducted all procedures following a consistent protocol and under uniform conditions. Briefly, 2 mL of blood samples were placed in an Eppendorf tube and permitted to coagulate naturally at room temperature for a duration of 2 h. Serum samples were subsequently isolated through centrifugation at 3,000 g for 15 min at 4°C. Hemolytic samples were discarded because the protein content in red blood cells could potentially influence proteomic analysis (27, 29). Subsequently, each serum sample was aliquoted, mixed with a protease inhibitor (Halt Protease Inhibitor Cocktail, Thermo Scientific, MA, United States) and a phosphatase inhibitor (sodium orthovanadate: Na_3_OV_4_, Sigma-Aldrich, MA, United States) to prevent protein degradation and dephosphorylation, and then stored at -20°C until assay.

### Analysis of serum proteins by LC–MS/MS

Serum samples that had been frozen were thawed at room temperature immediately before analysis. No serum sample was thawed more than once. The total protein concentration in each serum sample was assessed using the Lowry method, with bovine serum albumin serving as the protein standard. Each serum sample was processed to a final concentration of 0.25 μg/μl in 10 mM ammonium bicarbonate. Serum samples were exposed to 10 mM dithiothreitol in 10 mM ammonium bicarbonate and allowed to incubate at 56°C for 1 h to break down disulfide bond. Subsequently, cysteine residues were alkylated by adding 100 mM iodoacetamide in 10 mM ammonium bicarbonate to serum samples and incubated in a light-protected environment at room temperature for 1 h. Then, proteins were subjected to digestion by the addition of trypsin (Trypsin, Mass Spec Grade, Promega, WI, United States) at a ratio of 1: 20 (w/w) to the serum samples and kept for 3 h at ambient temperature. Afterward, serum samples were made acidic by the addition of 1% formic acid to stop enzymatic digestion. All peptide samples were introduced into an Ultimate3000 Nano/Capillary LC System (Thermo Scientific, MA, United States) connected to a Hybrid quadrupole Q-Tof impact II™ (Bruker Daltonics, MA, United States) that featured a Nano-captive spray ion source (30). Briefly, peptides underwent an enrichment step using a μ-Precolumn (300 μm i.d. × 5 mm) packed with C18 Pepmap 100 (5 μm, 100 A) (Thermo Scientific, MA, United States), and were separated on a column (75 μm I.D. × 15 cm) filled with Acclaim PepMap RSLC C18 (2 μm, 100 Å, nanoViper) (Thermo Scientific, MA, United States). The C18 column was maintained at a constant temperature of 60°C within a column oven. Solvent A and B containing 0.1% formic acid in water and 0.1% formic acid in 80% acetonitrile, respectively, were introduced into the analytical column. A gradient ranging from 5 to 55% solvent B was employed to elute the peptides, maintaining a constant flow rate of 0.30 μL/min over a 30 min duration. Electrospray ionization was carried out at 1.6 kV using the CaptiveSpray system, with nitrogen serving as the drying gas at a flow rate of around 50 L/h. Collision-induced-dissociation (CID) product ion mass spectra were produced using nitrogen as the collision gas. Mass spectra (MS) and MS/MS spectra were acquired in positive-ion mode at a frequency of 2 Hz, covering the range of *m/z* 150–2,200. The collision energy was tuned into 10 eV with response to the *m*/*z* value. To evaluate the stability of the mass spectrometry instrument, the bovine serum albumin digestion was employed as a quality control sample, integrated every 10 samples. This served to assess the performance of mass spectrometry by monitoring the consistency of quality control samples throughout various runs and the entire analytical workflow (31–33). Each serum sample was run in 3 replicates, and the mean intensity was used as final measurement to identify differential protein expression among groups by using the web-based tool Metaboanalyst (version 5.0; RRID: SCR_015539) (34, 35).

MaxQuant (version 1.6.6.0; RRID:SCR_014485) was employed to extract the proteins quantities in each sample by utilizing the raw data obtained from LC–MS/MS (36). The MS/MS spectra were correlated with the Uniprot *Canis lupus familiaris* database using the Andromeda search engine was utilized with a *p*-value of <0.05 and a false discovery rate (FDR) of 1% (37). The database was downloaded from UniProt on July 13, 2022. The process of label-free quantitation was executed using MaxQuant’s standard configuration, including parameters such as a maximum allowance of two missed cleavages, a main search mass tolerance of 0.6 daltons, trypsin as the enzyme for digestion, fixed modification of cysteine through carbamidomethylation, and variable modifications for methionine oxidation and protein N-terminus acetylation. Peptides were considered for identification and subsequent data analysis if they had a minimum length of 7 amino acids and at least one unique peptide. The single peptide approach was chosen for use in this study for potentially identify a larger set of proteins (38–39). In a subsequent step, the identified proteins in each group were plotted using a Venn diagram (RRID: SCR_016343). Exclusive quantification of within each group and common quantification among groups were then determined. The web-based tool MetaboAnalyst (version 5.0; RRID:SCR_015539) was used for statistical evaluation to identify differentially expressed proteins among groups. To enhance the reliability of the subsequent analysis on differential expression, only proteins detected in at least 60% of samples in at least one group were considered further (40). The biological processes of the differentially expressed proteins were analyzed using UniProt resources (Release 2022_03; RRID:SCR_002380) and PANTHER (RRID:SCR_004869). Finally, the online-based software Stitch (version 5.0; RRID:SCR_007947) was used to evaluate the relationship between differentially expressed proteins and commonly used cardiovascular drugs.

### Statistical analysis

The clinical features of the dogs were presented in the form of descriptive statistics. The statistical software (SPSS, RRID:SCR_002865) was used for assessing the statistically significant difference of the data. The Shapiro–Wilk test was utilized to assess normality. For the data that exhibited a normal distribution, the mean and standard deviation (SD) were provided. To compare the control, MMVD and MMVD+PH groups, a one-way analysis of variance (ANOVA) was performed, followed by post-hoc analysis utilizing the Bonferroni test. Statistical significance was defined as a *p*-value below 0.05. The data of identified serum proteins data were uploaded to the web-based tool MetaboAnalyst (version 5.0; RRID:SCR_015539) for statistical evaluation. To illustrate the distinction between various groups of identified proteins, partial least squares-discriminate analysis (PLS-DA) was utilized. Additionally, an ANOVA test, followed by Tukey’s *post hoc* test, was conducted to identify the differentially expressed proteins among groups (*p* < 0.05, FDR <0.05). Interested proteins were selected if they showed at least a 2-fold significant difference between MMVD+PH and MMVD groups.

## Results

### Clinical characteristic of dogs

Eighty-one dogs owned by clients participated in this study and were categorized into 3 groups: the control (*n* = 28), MMVD (*n* = 24), and MMVD+PH groups (*n* = 29). The clinical characteristics of the dogs are shown in Table 1. The ages of the dogs in the MMVD and MMVD+PH groups did not exhibit significant differences, but these dogs were notably older than those in the control group. There was no significant difference in body weight among the groups. The number of male and female dogs in the control and MMVD+PH groups was approximately equal, but the MMVD group consisted mainly of male dogs (Table 1).

**Table 1:** Clinical characteristics of dogs in the control, myxomatous mitral valve disease (MMVD) and myxomatous mitral valve disease with pulmonary hypertension (MMVD+PH) groups.

Parameters	Control (*n* = 28)	MMVD (*n* = 24)	MMVD + PH (*n* = 29)	*p*
Age (years)	9.14 ± 2.16	11.33 ± 1.97^a^	11.97 ± 2.37^a^	<0.001
Body weight (kg)	5.55 ± 2.30	5.13 ± 1.73	5.23 ± 1.57	0.697
Sex Male Female	12 16	19 5	13 16	
Breed	Shih-tzus (*n* = 11), Chihuahuas (*n* = 6), Poodles (*n* = 4), Pomeranians (*n* = 4), Yorkshire terriers (*n* = 3)	Poodles (*n* = 7), Pomeranians (*n* = 7), Chihuahuas (*n* = 6), Miniature pinchers (*n* = 2), Shih-tzus (*n* = 1), Mixed breed (*n* = 1)	Poodles (*n* = 8), Chihuahuas (*n* = 8), Shih-tzus (*n* = 4), Miniature pinchers (*n* = 3), Pomeranians (*n* = 2), Mixed breeds (*n* = 2), Jack russel terrier (*n* = 1), Schnauzer (*n* = 1)	

Data are reported as mean ± standard deviation (SD).

The significant difference was determined through one-way ANOVA at significance level of *p* < 0.05.

^a^Significant difference was observed at *p* < 0.05 when compared to the control group.

### History taking and physical examination

Data from the history and physical examination are presented in Table 2. There was no history or clinical indication of cardiorespiratory disease in the dogs in the control group. All dogs in the MMVD and MMVD+PH groups displayed clinical signs of congestive heart failure (CHF), including cough, exercise intolerance and dyspnoea. Dogs in the MMVD+PH group had clinical findings strongly suggestive of PH, including syncope and right-sided heart failure (ascites). Physical findings of dogs in the MMVD and MMVD+PH groups revealed heart murmurs, crackles and increased lung sounds, pale pink mucous membranes and a distended abdomen. There were no significant variations in heart rate and blood pressure among the groups (Table 2). All dogs in the MMVD and MMVD+PH groups exhibited stable clinical signs of heart failure and had been receiving standard cardiovascular medications such as angiotensin-converting enzyme inhibitors (ACEIs), diuretics, inotropics and vasodilators for more than 1 month. In the control group, no dog had been receiving medication for at least 1 month prior to blood sampling.

**Table 2:** History taking and physical examination data of dogs in the control, myxomatous mitral valve disease (MMVD) and myxomatous mitral valve disease with pulmonary hypertension (MMVD+PH) groups.

Parameters	Control (*n* = 28)	MMVD (*n* = 24)	MMVD + PH (*n* = 29)	*p*
Clinical signs Cough Exercise intolerance Respiratory distress Syncope Right-sided CHF (ascites)	– – – – –	24 (100%) 18 (75%) 6 (25%) – –	29 (100%) 23 (79.31%) 8 (27.59%) 5 (17.24%) 7 (24.14%)	
Physical examination Systolic heart murmur Crackle lung sound Increased lung sound Pale pink mucous membrane Abdominal distension	– – – – –	24 (100%) 2 (8.33%) 13 (54.17%) 1 (4.17%) –	29 (100%) 5 (17.24%) 18 (62.07%) 10 (34.48%) 7 (24.14%)	
Heart rate (bpm)	129.43 ± 16.87	133.63 ± 27.04	137.10 ± 25.05	0.462
Blood pressure (mmHg)	144.00 ± 18.36	138.33 ± 20.25	134.24 ± 19.31	0.166
Medication ACEIs Furosemide Pimobendane Spironolactone Amirolide and hydrochlorothiazide (Moduretic®) Sildenafil	– – – – – –	24 (100%) 24 (100%) 24 (100%) 9 (37.5%) 6 (25%) –	29 (100%) 29 (100%) 29 (100%) 12 (41.38%) 7 (24.14%) 3 (10.34%)	

Data are reported as mean ± standard deviation (SD) and the proportion (percent).

The significant difference was determined through one-way ANOVA at significance level of *p* < 0.05.

### Routine hematology and blood chemistry profiles

The routine hematologic and blood chemistry assessments of all dogs in this study fell within the expected or healthy range (Table 3). However, when comparing between groups, red blood cells, haemoglobin and haematocrit were significantly decreased, while white blood cells and neutrophils were significantly elevated in the MMVD+PH group in contrast to the control and MMVD groups. Blood chemistry profiles revealed significant increases in alanine aminotransferase, alkaline phosphatase and blood urea nitrogen in both the MMVD and MMVD+PH groups in comparison to the control group. Other variables showed no significant differences among the groups (Table 3). Point-of-care test kits (SNAP4Dx, IDEXX Laboratories, ME, United States) showed that all dogs in this study were negative for heartworm antigen and blood parasite infections including ehrlichiosis, anaplasmosis and Lyme disease.

**Table 3:** Complete blood count and blood chemistry profiles of dogs in the control, myxomatous mitral valve disease (MMVD) and myxomatous mitral valve disease with pulmonary hypertension (MMVD+PH) groups.

Parameters	Control (*n* = 28)	MMVD (*n* = 24)	MMVD + PH (*n* = 29)	*p*
RBC (x10^6^ cell/μL)	6.88 ± 0.84	6.96 ± 0.78^b^	6.17 ± 1.14^a,c^	0.005
Hb (g/dL)	16.84 ± 2.00	16.17 ± 1.75^b^	14.65 ± 2.55^a,c^	0.001
Hct (%)	43.95 ± 4.76	43.57 ± 5.35^b^	39.57 ± 6.33^a,c^	0.006
MCV (fL)	63.88 ± 2.80	64.60 ± 5.21	62.64 ± 3.64	0.217
MCH (pg)	24.13 ± 1.11	23.84 ± 1.69	23.26 ± 1.45	0.072
MCHC (g/dL)	38.35 ± 0.91	38.30 ± 5.54	36.98 ± 1.46	0.195
Platelet (x10^3^cell/μL)	310.36 ± 90.56	332.88 ± 94.21	363.90 ± 101.11	0.111
WBC (x10^3^cell/uL)	8.86 ± 3.26	9.75 ± 2.47^b^	12.60 ± 3.96^a,c^	<0.001
Neutrophils (x10^3^cell/uL)	6.08 ± 2.90	7.03 ± 2.06 ^b^	9.42 ± 3.93^a,c^	<0.001
Eosinophils (x10^3^cell/uL)	0.44 ± 0.38	0.57 ± 0.51	0.50 ± 0.34	0.514
Basophils (x10^3^cell/uL)	0.02 ± 0.01	0.02 ± 0.01	0.02 ± 0.01	0.504
Lymphocytes (x10^3^cell/uL)	1.78 ± 0.90	1.34 ± 0.42	1.57 ± 0.82	0.117
Monocytes (x10^3^cell/uL)	0.67 ± 0.50	0.78 ± 0.29	0.86 ± 0.31	0.172
ALT (IU/L)	42.29 ± 16.44	67.13 ± 38.15^a^	63.65 ± 42.78^a^	0.019
ALP (IU/L)	57.21 ± 40.46	109.29 ± 84.79^a^	116.62 ± 93.60^a^	0.009
Creatinine (mg/dL)	0.89 ± 0.12	0.96 ± 0.23	0.99 ± 0.20	0.112
BUN (mg/dL)	17.45 ± 6.60	33.06 ± 15.62^a^	38.49 ± 19.59^a^	<0.001
Total protein (g%)	6.50 ± 1.22	6.83 ± 0.54	6.30 ± 1.21	0.196
Albumin (g%)	3.14 ± 0.34	3.43 ± 0.51	3.14 ± 0.54	0.051

RBC, Red blood cell; Hb, Hemoglobin; Hct, Hematocrit; MCV, Mean corpuscular volume.

MCH, Mean corpuscular hemoglobin; MCHC, Mean corpuscular hemoglobin concentration; WBC, White blood cell; ALT, Alanine aminotransferase; ALP, Alkaline phosphatase; BUN, Blood urea nitrogen.

Data are reported as mean ± standard deviation (SD).

The significant difference was determined through one-way ANOVA at significance level of *p* < 0.05.

^a^Significant difference was observed at *p* < 0.05 when compared to the control group.

^b,c^Significant difference was observed at *p* < 0.05 when comparing between the MMVD and MMVD + PH groups.

### Electrocardiography, thoracic radiography and echocardiography

Electrocardiography showed that all dogs in the control group and almost all dogs in the MMVD (23/24) and MMVD+PH (21/29) groups displayed respiratory sinus arrhythmia, whereas the others showed sinus rhythm (Table 4).

**Table 4:** Electrocardiogram, thoracic radiographic findings and echocardiographic indices of dogs in the control, myxomatous mitral valve disease (MMVD) and myxomatous mitral valve disease with pulmonary hypertension (MMVD+PH) groups.

Parameters	Control (*n* = 28)	MMVD (*n* = 24)	MMVD + PH (*n* = 29)	*p*
Electrocardiogram				
Respiratory sinus arrhythmia	28 (100%)	23 (95.83%)	21 (72.41%)	
Sinus rhythm	–	1 (4.17%)	8 (27.59%)	
Thoracic radiography				
VHS	9.54 ± 0.77	11.67 ± 0.87^a^	12.21 ± 0.96^a^	<0.001
Left atrial enlargement Pulmonary vein congestion Pulmonary edema	– – –	24 (100%) 6 (25%) 6 (25%)	29 (100%) 10 (34.48%) 10 (34.48%)	
Pulmonary artery enlargement	–	–	9 (31.03%)	
Right-sided heart enlargement	–	–	9 (31.03%)	
Echocardiography				
IVSd	0.49 ± 0.10	0.47 ± 0.06	0.46 ± 0.10	0.477
LVIDd	1.22 ± 0.13	1.81 ± 0.26^a^	1.72 ± 0.43^a^	<0.001
LVPWd	0.39 ± 0.07	0.41 ± 0.08	0.41 ± 0.11	0.668
IVSs	0.63 ± 0.1	0.65 ± 0.11	0.66 ± 0.16	0.663
LVIDs	0.71 ± 0.13	0.95 ± 0.18^a^	0.87 ± 0.37^a^	0.004
LVPWs	0.65 ± 0.10	0.67 ± 0.11	0.71 ± 0.19	0.210
LA index	0.85 ± 0.14	1.99 ± 0.59^a^	1.90 ± 0.75^a^	<0.001
AO index	0.74 ± 0.09	0.81 ± 0.32	0.89 ± 0.31	0.093
LA/AO	1.15 ± 0.16	2.00 ± 0.41^a^	2.17 ± 0.60^a^	<0.001
%FS	39.01 ± 8.33	45.86 ± 8.00^a^	51.22 ± 10.81^a^	<0.001
PAP (mmHg)	–	–	61.13 ± 21.51	–

The echocardiographic indices were normalized by using Cornell’s allometric scaling method.

VHS, Vertebral heart score; IVSd, Interventricular septal thickness at end diastole; LVIDd, Left ventricular internal diameter at end diastole; LVPWd, Left ventricular posterior wall thickness at end diastole; IVSs, Interventricular septal thickness at end systole; LVIDs, Left ventricular internal diameter at end systole; LVPWs, Left ventricular posterior wall thickness at end systole; LA, Left atrium; AO, Aorta; LA:AO, Left atrium to aorta ratio; FS, fractional shortening; PAP, pulmonary arterial pressure.

Data are reported as mean ± SD and the proportion (percent).

The significant difference was determined through one-way ANOVA at significance level of *p* < 0.05.

^a^Significant difference was observed at *p* < 0.05 when compared to the control group.

Thoracic radiography revealed that dogs in the control group had normal radiographic findings, but all dogs in the MMVD and MMVD+PH groups were diagnosed with cardiomegaly with a vertebral heart score (VHS) of >10.5 with left atrial enlargement. The VHS of dogs in the MMVD and MMVD+PH groups was significantly higher than in the control group but there was no statistically significant difference in VHS between the MMVD and MMVD+PH groups. All MMVD dogs, both with and without PH, had pulmonary oedema as assessed by thoracic radiography more than 1 month before participation in this study. Six of 24 dogs in the MMVD group and 10 of 29 dogs in the MMVD+PH group had pulmonary vein congestion and pulmonary oedema on the day of blood sampling. In the MMVD+PH group, 9 of 29 dogs had PA enlargement and right-sided heart enlargement (Table 4).

Echocardiographic findings revealed unremarkable cardiac abnormalities in the control group, whereas enlargement of the left atrium and left ventricle was noted in all MMVD dogs with and without PH. The left atrial diameter (LA), the left atrial to aorta dimension ratio (LA/AO), the left ventricular internal diameter at the end of diastole (LVIDd), the left ventricular internal diameters at the end of systole (LVIDs) and fractional shortening (%FS) were significantly greater in the MMVD and MMVD+PH groups than in the control group. When comparing the MMVD and MMVD+PH groups, none of the echocardiographic indices showed significant difference (Table 4). On the basis of the estimated PAP and anatomic signs of PH, almost all PH dogs secondary to MMVD were identified with a high probability of PH (22/29); the others were diagnosed as having an intermediate probability of PH (7/29). The MMVD dogs with a high probability of PH exhibited significantly elevated values for the peak TR velocity and estimated PAP (4.05 ± 0.60 m/s; 67.16 ± 21.37 mmHg) compared to dogs with an intermediate probability of PH (3.25 ± 0.11 m/s; 42.17 ± 2.84 mmHg) (*p* < 0.001). No MMVD dog with an intermediate probability of PH had right-sided heart enlargement, whereas 14 of 22 MMVD dogs with a high probability of PH had right atrial and right ventricular enlargement (Table 5).

**Table 5:** The peak tricuspid regurgitation (TR) velocity and estimated pulmonary arterial pressure (PAP) and anatomic signs of pulmonary hypertension (PH) of myxomatous mitral valve disease (MMVD) dogs with intermediate and high probability of PH.

Parameters	Intermediate probability of PH (*n* = 7)	High probability of PH (*n* = 22)	*p*
Peak TR velocity (m/s)	3.25 ± 0.11	4.05 ± 0.60^a^	<0.001
PAP (mmHg)	42.17 ± 2.84	67.16 ± 21.37^a^	<0.001
Anatomic sites of echocardiographic signs of PH Ventricles Pulmonary artery Right atrium and caudal vena cava	– 6 (85.71%) –	14 (63.64%) 22 (100%) 14 (63.64%)	

Data are reported as mean ± SD and the proportion (percent).

The significant difference was determined through the independent-samples *T* Test at significance level of *p* < 0.05.

^a^Significant difference was observed at *p* < 0.05 when comparing between MMVD with intermediate and high probability of PH groups.

### Protein identification by LC–MS/MS

**Figure 1:**
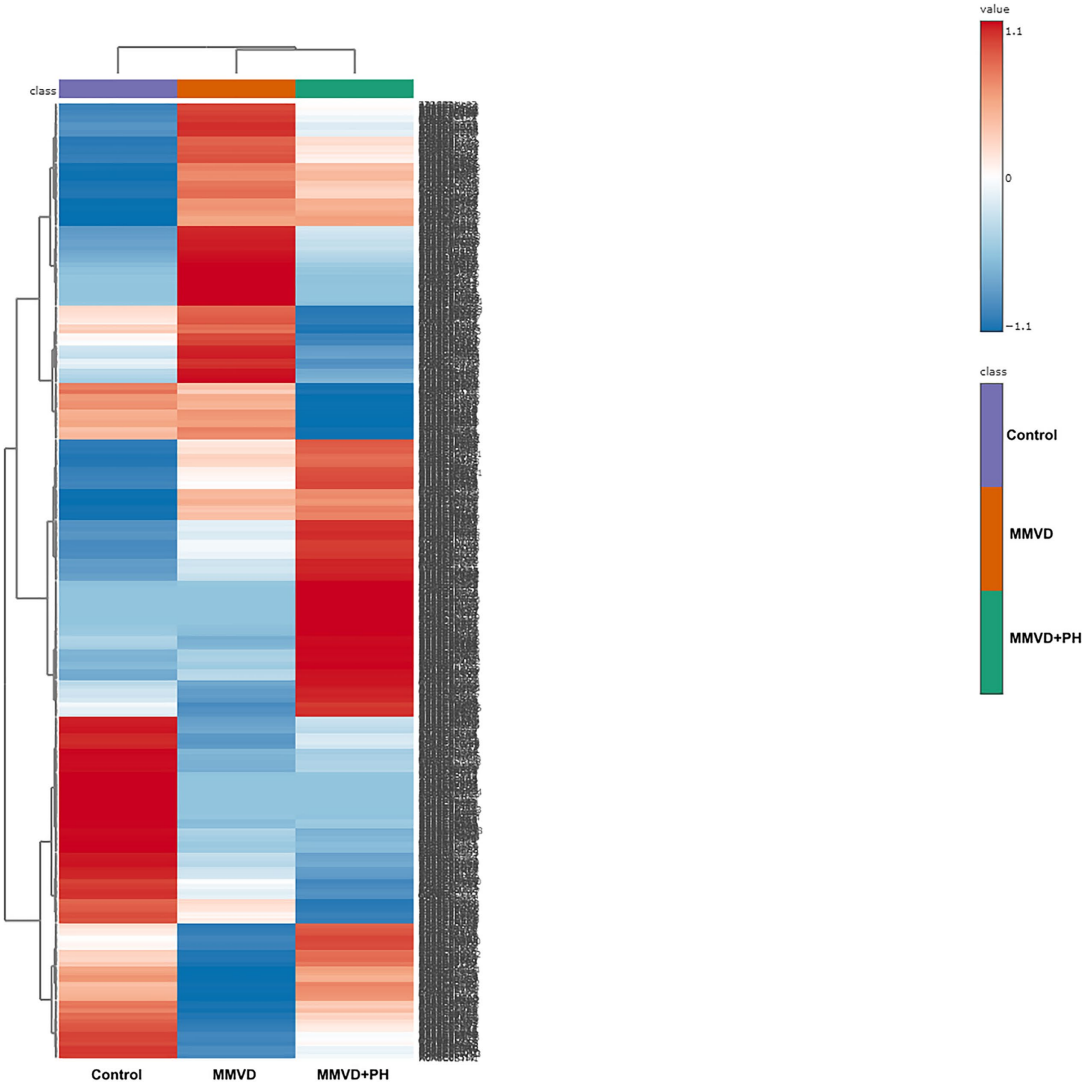
Heat map of all identified proteins in the control, myxomatous mitral valve disease (MMVD) and myxomatous mitral valve disease with pulmonary hypertension (MMVD+PH) groups. A total of 1,211 proteins were quantified, exhibiting varied expression levels across different groups. The groups are arranged in columns, while the identified proteins are listed in rows. Protein intensity is represented by color, ranging from very low (deep blue) to extremely high (dark brown), eith the color scale on the right indicating the range of expression values.

**Figure 2:**
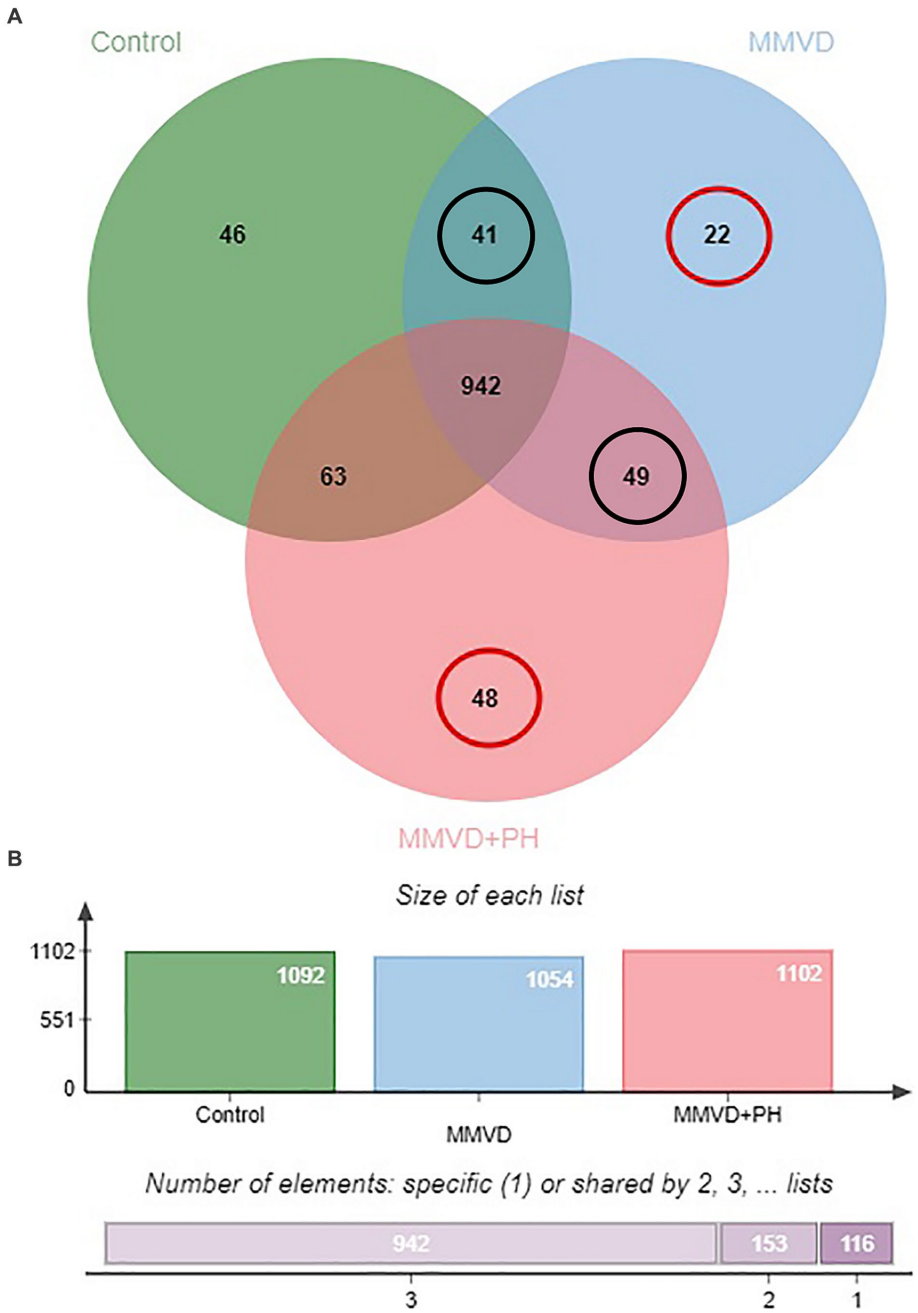
**(A)** Venn diagram of identified proteins showed overlap and uniquely found proteins in the control, myxomatous mitral valve disease (MMVD) and myxomatous mitral valve disease with pulmonary hypertension (MMVD+PH) groups. Twenty-two and 48 proteins were uniquely expressed in the MMVD and MMVD with PH groups, respectively. Forty-one proteins were commonly identified in both the control and MMVD groups, while 49 proteins were commonly identified in both MMVD and MMVD+PH groups. **(B)** The overall count of recognised proteins was 1,092, 1,054 and 1,102 for the control, MMVD and MMVD+PH groups, respectively.

**Figure 3:**
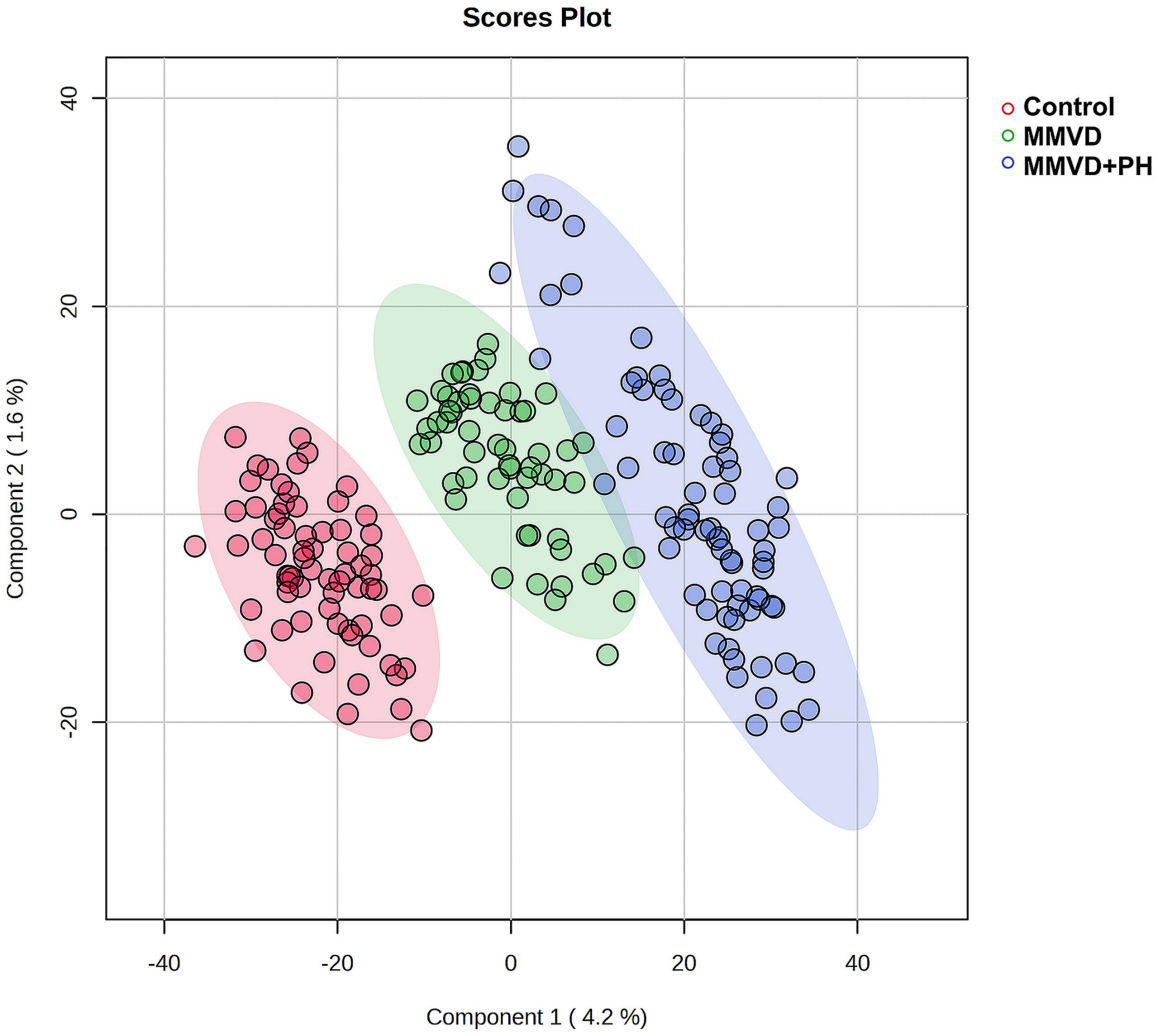
Partial least squares-discriminant analysis (PLS-DA) was conducted on all identified proteins, with samples clustered according to groups: the control, myxomatous mitral valve disease (MMVD) and myxomatous mitral valve disease with pulmonary hypertension (MMVD+PH) groups. The PLS-DA results demonstrated good separation of identified proteins among groups with minor overlap between the MMVD and MMVD+PH groups. Individual samples are represented by colored dots, while colored areas depict the 95% confidence interval.

**Figure 4:**
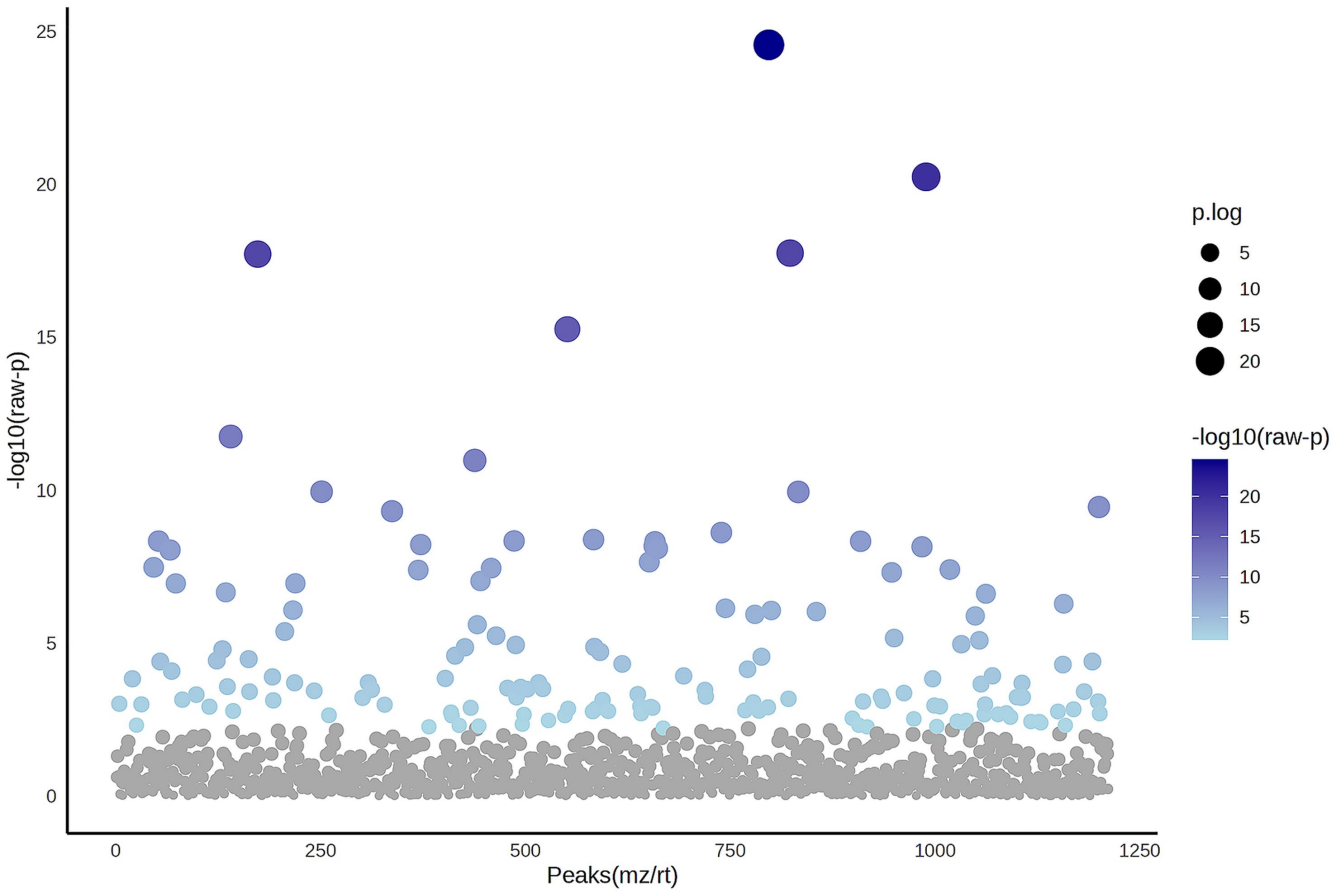
The Analysis of Variance (ANOVA) plot illustrates the comparison among the control, myxomatous mitral valve disease (MMVD) and myxomatous mitral valve disease with pulmonary hypertension (MMVD+PH) groups for significantly identified proteins. An ANOVA test identified 151 out of 1,211 proteins that exhibited significant differences among the groups. The blue dots indicate significantly expressed proteins with *p* < 0.05, while grey dots represent proteins without statistical significance.

**Figure 5:**
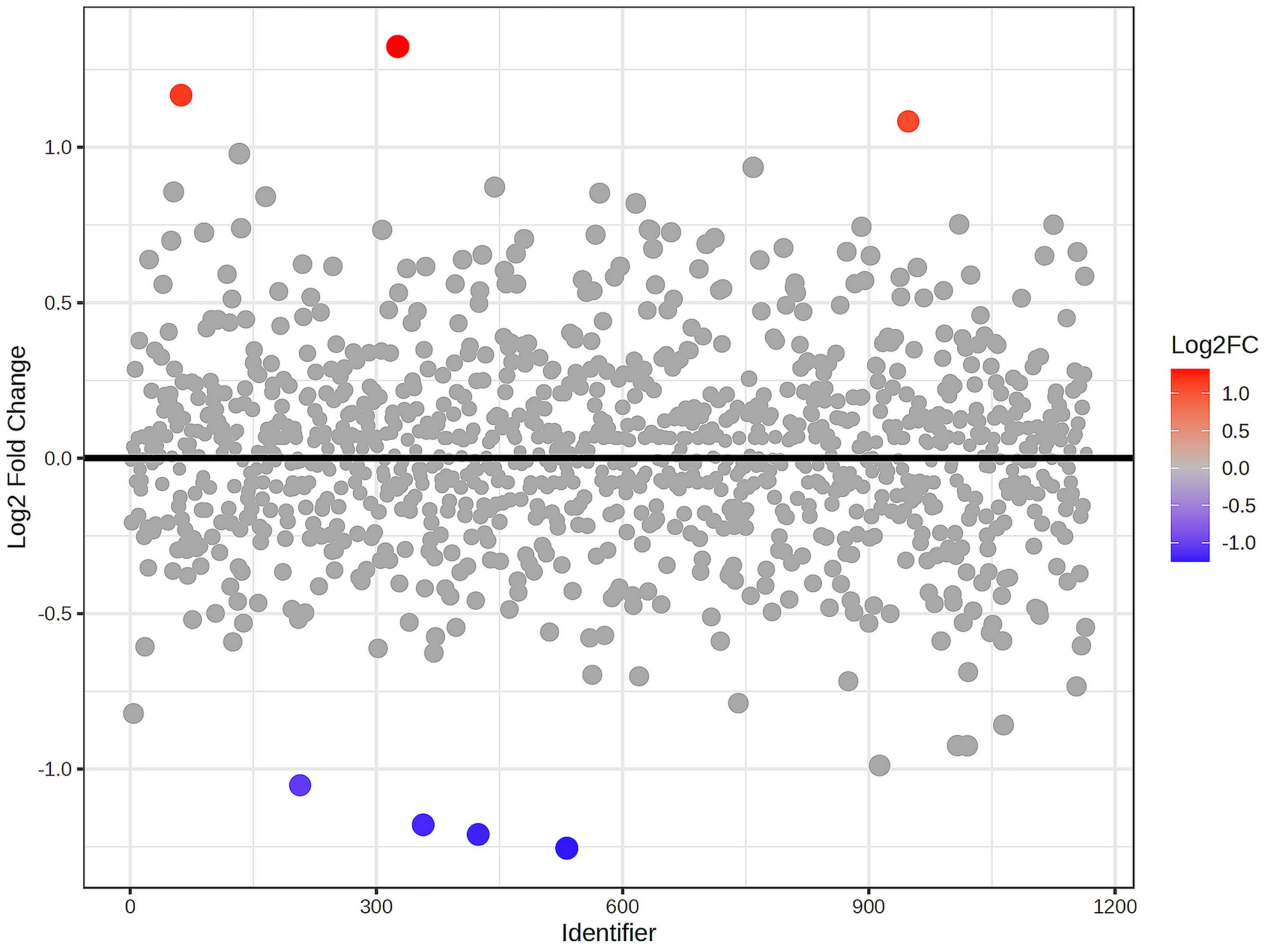
Fold change analysis of identified proteins comparing the myxomatous mitral valve disease with pulmonary hypertension (MMVD+PH) and myxomatous mitral valve disease (MMVD) groups. The fold change values represent the ratio expression levels between the groups, where positive values denote upregulation and negative values denote downregulation. The scatter plot visualises the log2-transformed fold changes in protein abundance between groups. Gray dots represent proteins with fold change <2, while the colored dots represent significantly upregulated (red dots) and downregulated (blue dots) proteins with fold change >2 and *p* < 0.05. The color intensity reflects the magnitude of the fold change.

**Figure 6:**
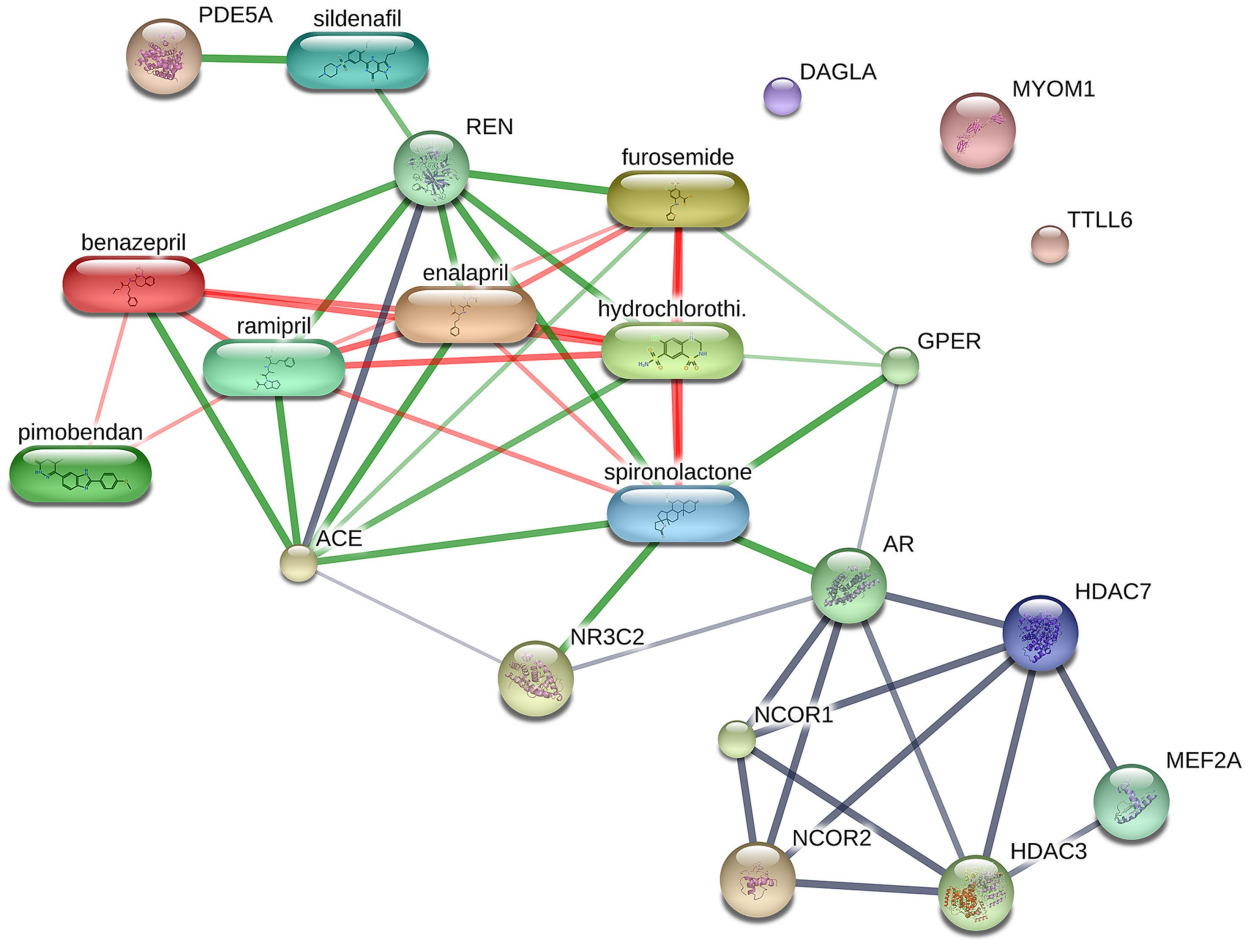
Network of protein–protein and protein-cardiovascular drugs interaction analysed by Stitch, version 5.0. The selected upregulated and downregulated proteins in the myxomatous mitral valve disease with pulmonary hypertension (MMVD+PH) groups including Myomesin 1 (MYOM1), Diacylglycerol lipase alpha (DAGLA) and Tubulin tyrosine ligase like 6 (TTLL6) showed no correlation with any cardiovascular drugs, whereas Histone deacetylase 7 (HDAC7) showed a weak correlation with spironolactone. The strength of the associations at the functional level was evaluated by edge confidence scores. The strong relationships with high edge confidence scores (>0.700) are presented as thick lines.

A total of 1,211 proteins were identified, as shown in the heat map (Figure 1). The overall count of recognized proteins was 1,092, 1,054 and 1,102 for the control, MMVD and MMVD+PH groups, respectively. The Venn diagram showed that 22 proteins were expressed only in the MMVD group, while 48 proteins were expressed only in the MMVD+PH group. Forty-one proteins were commonly identified in both the control and MMVD groups, while 49 proteins were commonly identified in both MMVD and MMVD+PH groups (Figure 2). The partial least squares-discriminate analysis (PLS-DA) illustrated good separation of identified proteins among groups with minor overlap between the MMVD and MMVD+PH groups (Figure 3). Based on the database search, 1,035 peptides were detected as part of identified proteins, whereas the remaining 176 peptides represented uncharacterised proteins. The ANOVA test with Tukey’s *post hoc* analysis revealed that 151 out of 1,211 identified proteins showed significant difference (*p* < 0.05, FDR < 0.05) among the control, MMVD and MMVD+PH groups (Figure 4). The proteins that exhibit fold change greater than 2 in the MMVD+PH compared to the MMVD groups were focused. The top 3 upregulated and downregulated proteins were selected as interested proteins. There were 7 proteins (3 upregulated, 4 downregulated) with fold changes greater than 2 in the MMVD+PH compared to the MMVD groups (Figure 5). Among 7 differentially expressed proteins, 2 proteins represented uncharacterised proteins. The remaining 2 upregulated proteins were identified as Myomesin 1 (MYOM1) and Histone deacetylase 7 (HDAC7) proteins, while the remaining 3 downregulated proteins were identified as Pleckstrin homology domain containing M3 (PLEKHM3), Diacylglycerol lipase alpha (DAGLA), and Tubulin tyrosine ligase like 6 (TTLL6) (Table 6). According to the proteins functional analysis and pathway search conducted by PANTHER, unfortunately, only one protein, PLEKHM3 was classified to be associated with cellular process, while the remaining proteins were unclassified by PANTHER. The protein–protein interaction networks and the interactions between proteins with commonly used cardiovascular drugs are shown in Figure 6. The selected proteins, including MYOM1, TTLL6 and DAGLA, showed no correlation with any cardiovascular drugs, whereas HDAC7 showed a weak correlation with spironolactone (Figure 6). Unfortunately, the STITCH database did not find PLEKHM3 in *Canis lupus*. Proteins detected in at least 60% of samples in at least one group, including MYOM1 (79.3% in MMVD+PH group), HDAC7 (89.66% in MMVD+PH group), and DAGLA (62.5% in MMVD group), were selected as interested proteins.

**Table 6:** The upregulated and downregulated proteins in the serum of dogs in the myxomatous mitral valve disease with pulmonary hypertension (MMVD+PH) group compared to dogs in the myxomatous mitral valve disease (MMVD) group.

Protein name	Fold change	*p*
Downregulation		
Pleckstrin homology domain containing M3 (PLEKHM3)	0.41915	<0.001
Diacylglycerol lipase alpha (DAGLA)	0.43217	<0.001
Uncharacterised protein Tubulin tyrosine ligase like 6 (TTLL6)	0.44152 0.48227	<0.001 <0.001
Upregulation		
Histone deacetylase 7 (HDAC7)	2.1188	<0.001
Myomesin 1 (MYOM1)	2.2462	<0.001
Uncharacterised protein	2.5036	<0.001

The significant difference was determined through Tukey’s post hoc analysis at significance level of *p* < 0.05.

## Discussion

This study investigated the expression of proteins in serum by LC–MS/MS in healthy control dogs and MMVD dogs with and without PH. The findings strongly supported our hypothesis, revealing significant differences in protein signaling intensities among MMVD dogs with PH, those without PH, and healthy control dogs. The primary discovery of this study is the identification, through LC–MS/MS, differentially expressed proteins that may contribute to the pathogenesis of PH in MMVD dogs. Previously, biomarker discovery was done by selecting proteins of interest and then measuring them using specific methods such as enzyme-linked immunosorbent assay (ELISA). More recently, proteomic methods such as LC–MS/MS has been developed and employed for the identification of potential biomarker candidates without the need to know the proteins of interest in advance, as a variety of proteins can be detected and identified in samples (41). Although several studies have reported potential protein biomarkers in people with PH (20–22), the results have been inconclusive. The results obtained by different laboratories are not comparable or reproducible, probably because there is still a lack of standardized protocols for the preanalytical procedures that affect the sensitivity, selectivity, reproducibility and the recovery of reliable biomarkers (28). Recently, recommendations for appropriate proteomic analysis in biomarker studies have been proposed (27, 28, 42). Sample collection and preparation in this study were performed by an investigator using the same protocol and conditions as recommended.

MMVD stands as the common heart disease in older small-breed dogs (43). The likelihood of small-breed dogs developing MMVD rises with advancing age, as reported by Pascon et al. (44). The prevalence of MMVD was 23.9% in dogs aged 6–10 years and increased to 30.3% in dogs aged 10–19 years. Because MMVD occurs primarily in older small-breed dogs (2, 25), age- and weight-matched dogs were selected to reduce the effects of age and breed differences on serum protein expression. Unfortunately, the control group’s average age was significantly younger compared to the age of the dogs in the MMVD and MMVD+PH groups. It is difficult to include healthy aging dogs in the control group because older dogs usually have MMVD or other diseases such as chronic kidney disease or cancer. However, all dogs in this study were in the same age group considered to be older dogs (>7–10 years) (45). The number of male and female dogs was roughly equal in the control and MMVD+PH groups, but the MMVD group was mainly composed of male dogs. The sex differences between the groups may have affected the findings from the study (46, 47).

According to the ACVIM consensus guidelines for the diagnosis and treatment of myxomatous mitral valve disease in dogs (25), all dogs with MMVD stage C included in this study had signs of heart failure and required treatment with cardiovascular medications. To minimize the effects of the drugs on serum protein expression, all dogs in the MMVD and MMVD+PH groups had to be managed with standard cardiovascular medications including ACEIs, furosemide and pimobendan for more than 1 month, whereas dogs in the control group could not receive any medications for at least 1 month before blood collection. However, some dogs in the MMVD and MMVD+PH groups also received other medications such as a combination of diuretic drugs, amiloride and hydrochlorothiazide (moduretic®), spironolactone and sildenafil which may have affected serum protein expression (48–50).

Several investigations have reported alterations in serum protein expression in individuals with systemic diseases such as kidney disease (51, 52), liver disease (53–55) and blood parasite infections (56–59). In the present study, routine haematologic and blood chemistry profiles were evaluated to exclude dogs with systemic diseases that might affect serum protein expression. Although the blood profiles of all dogs were within normal limits, some values differed between groups. Haematological profiles showed that RBC, Hb and Hct decreased significantly; whereas WBC and neutrophils were significantly elevated in the MMVD+PH group in comparison to the other groups. Decreases in RBC, Hb and Hct have been observed in people with heart failure, possibly because of chronic disease (60). The relationship between heart failure and inflammation in people has been described previously (61, 62). There were no dogs with heart failure in the present study whose WBC and neutrophils exceeded the normal range. However, the MMVD+PH group had higher WBC and neutrophils than the other groups. This finding is inconsistent with previous studies showing an elevation in WBC and neutrophil counts in dogs experiencing advanced stage or decompensated heart failure (63, 64). Liver enzymes and BUN were elevated in the MMVD and MMVD+PH groups as opposed to the control group. An increase in liver enzymes could be due to CHF induced liver injury caused by heart failure, whereas an increase in BUN could be due to impaired renal function or diuretic use.

This study has demonstrated that LC–MS/MS has the potential to be used to identify interested proteins that are differentially expressed in MMVD dogs with PH compared to those without PH. Techniques for prioritising candidates are often based on statistical significance and fold-change, their association with signalling pathways that change in disease, and correlation with published literature (65, 66). Because the diagnosis of MMVD is simple and straightforward, but the distinction between MMVD dogs with and without PH remains difficult, this study focused on the differential protein expression between MMVD dogs with and without PH. In this study, the interested proteins were selected based on fold change and *p*-value of *post hoc* analysis. Seven differentially expressed proteins (3 upregulated, 4 downregulated) demonstrated fold changes greater than 2 in the MMVD+PH compared to the MMVD groups. Of these, 2 proteins represented uncharacterised proteins, and the remaining 2 upregulated proteins were identified as Myomesin 1 (MYOM1) and Histone deacetylase 7 (HDAC7) proteins while the remaining 3 downregulated proteins were identified as Pleckstrin homology domain containing M3 (PLEKHM3), Diacylglycerol lipase alpha (DAGLA) and Tubulin tyrosine ligase like 6 (TTLL6). Interest proteins were then selected based on the proteins detected at least 60% of samples in at least one group, including MYOM1, HDAC7 and DAGLA were selected as interested proteins.

Myomesin 1 (MYOM1) is a protein primarily found in cardiac and skeletal muscles, playing a vital role in muscle structure and function. Located within the cytoplasm of myocytes, MYOM1 is a crucial component of sarcomere, the basic contractile unit of muscles, contributing to the structural organization and stability of muscle fibers (67–69). Although primarily known for its role in muscle structure, MYOM1 has been implicated in cardiovascular diseases, particularly hypertrophic cardiomyopathy (HCM) where mutations in the MYOM1 gene have been associated with abnormal thickening of the heart muscle (70). Overexpression of MYOM1 in myocardium has also been reported in patients with tetralogy of Fallot, potentially linked to impaired cardiac function (71). While direct connections between MYOM1 and PH are not yet established, ongoing research into the molecular mechanisms of PH underscores the significance of muscle proteins like titin in sarcomere organization and function (72). Further investigation is needed to fully elucidate MYOM1’s role in PH.

Histone deacetylase 7 (HDAC7) belongs to the histone deacetylase family, regulating gene expression by modifying chromatin structure. HDAC7 specifically removes acetyl groups from histone proteins, leading to chromatin condensation and transcriptional repression. In addition to its role in epigenetic regulation, HDAC7 also participates in diverse cellular processes, including cell cycle regulation, differentiation, and apoptosis, making it associated with various physiological and pathological conditions (73). Emerging evidence suggests a role for HDAC7 in the pathogenesis of PH (74). Overexpression of HDAC7 has been observed in the pulmonary arterial smooth muscle cells (PASMCs) within the medial layer of the pulmonary vasculature in human patients with PH. Pharmacological blocking and genetic elimination of HDAC7 result in induction of PASMCs apoptosis (75). In animal models, increased HDAC7 expression in the pulmonary artery of PH-induced rats was associated with elevated ROS production and Nox expression. Inhibition HDAC7 reversed fibrosis and inflammation, indicating its potential implication in pulmonary vascular remodeling and contribution to the development and progression of PH (76).

Diacylglycerol lipase alpha (DAGLA) is an enzyme in the endocannabinoid system, regulating various physiological processes by catalysing the hydrolysis of diacylglycerol (DAG) to produce the endocannabinoid 2-arachidonoylglycerol (2-AG). Endocannabinoids like 2-AG serve as signaling molecules, interacting with cannabinoid receptors to modulate neurotransmitter release and cellular functions (77). The endocannabinoid system, including DAGLA and its product 2-AG, may have implications for cardiovascular function. Research in rats has shown that intravenous injection of 2-AG induces hypotension and bradycardia (78). Elevated levels of 2-AG have been observed in plasma can cardiac tissue in myocardial infarction induced- mice, associated with increased DAGLA expression in infarcted hearts. Elevated 2-AG levels have been linked to impaired ventricular scar formation, worsened cardiac function, and increased neutrophil and monocyte counts (79). Additionally, 2-AG appears to impact atherogenesis, with mice showing increased plaque burden and macrophage infiltration within atherosclerotic vessels, suggesting a potential role in macrophage migration and vascular inflammation leading atherosclerosis (80). However, the involvement of DAGLA and 2-AG in PH requires further investigation.

This study concluded that LC–MS/MS has the potential to be used as a method to identify proteins of interest. Further research is required to validate the potential utility of these proteins as biomarkers.

There are three important constraints that must be acknowledged. To start with, this study did not include MMVD dogs with a low probability of PH because detection of PH at an early stage is difficult and usually incidental. In addition, the obvious clinical findings of PH are usually found in dogs with an estimated PAP greater than 48–55 mmHg (5, 81). Therefore, incorporating MMVD dogs with a low probability of PH into the study presents challenges. Second, serum protein expression could be influenced by several factors, such as age, sex and treatment with cardiovascular drugs, which cannot be completely controlled. Lastly, protein expression was not performed in this study. In recent years, the use of methods such as ELISA or western blot for validating proteomic data has been criticized. Concerns about antibody specificity and availability have been raised, and developing highly specific antibodies is a significant challenge. Differences in sample preparation between proteomics and other methods may contribute to varying results (82–84). Moreover, the lack of available antibodies for the targeted proteins in the context of this study is a significant issue. The major limitations of this study include the high cost and time-consuming nature in creating new antibodies, which, unfortunately, hindered the performance of a validation study.

## Data availability statement

The datasets presented in this study can be found in online repositories. The names of the repository/repositories and accession number(s) can be found in the article/Supplementary material.

## Ethics statement

The animal studies were approved by the Institutional Animal Care and Use Committee, Faculty of Veterinary Science, Chulalongkorn University (Approval number, 1831099; Approval date, 4 February 2019). The studies were conducted in accordance with the local legislation and institutional requirements. Written informed consent was obtained from the owners for the participation of their animals in this study.

## Author contributions

SSa: Formal analysis, Investigation, Project administration, Validation, Writing – original draft. AR: Writing – review & editing. SR: Conceptualization, Project administration, Validation, Writing – review & editing. JJ: Investigation, Writing – review & editing. NP: Investigation, Writing – review & editing. SC: Investigation, Writing – review & editing. ST: Investigation, Writing – review & editing. SSu: Conceptualization, Funding acquisition, Methodology, Project administration, Supervision, Writing – review & editing.

## Glossary

CHF: Congestive heart failure
DAGLA: Diacylglycerol lipase alpha
LC–MS/MS: Liquid chromatography–tandem mass spectrometry
MMVD: myxomatous mitral valve disease
MYOM1: Myomesin 1
HDAC7: Histone deacetylase 7
PA: pulmonary arteries
PAP: pulmonary arterial pressure
PLEKHM3: Pleckstrin homology domain containing M3
PH: pulmonary hypertension
TR: tricuspid regurgitation
TTLL6: Tubulin tyrosine ligase like 6
VHS: vertebral heart score
